# Exploring adolescents' experiences of continuing to wear face masks during COVID‐19: A qualitative descriptive study in Barcelona (Spain)

**DOI:** 10.1111/hex.14014

**Published:** 2024-03-13

**Authors:** Mariela Aguayo‐González, Juan M. Leyva‐Moral, David Giménez‐Diez, Andreu Colom‐Cadena, Isabel Martínez, Carolina Watson, Anna Bordas, Cinta Folch, Jordi Casabona

**Affiliations:** ^1^ Nursing Department, Faculty of Medicine Universidad Autónoma de Barcelona Bellaterra Spain; ^2^ Centre d'Estudis Epidemiològics sobre les ITS i Sida de Catalunya (CEEISCAT) Badalona Spain; ^3^ Institut d'Investigació Germans Trias i Pujol (IGTP) Badalona Spain; ^4^ Unitat de Suport a la Recerca Tarragona‐Reus, IDIAP Jordi Gol Tarragona Spain; ^5^ Centro de Investigación Biomédica en Red de Epidemiología y Salud Pública (CIBERESP) Instituto de Salud Carlos III Madrid Spain; ^6^ Departament de Pediatria, d'Obstetrícia i Ginecologia i de Medicina Preventiva i de Salut Publica, Institut d'Investigació Germans Trias i Pujol (IGTP) Universitat Autònoma de Barcelona Badalona Spain

**Keywords:** adolescents, COVID‐19, face masks, qualitative research

## Abstract

**Background:**

The COVID‐19 pandemic prompted the use of face masks as a social distancing measure. Although evidence supports their effectiveness in preventing infection, it remains unclear why some adolescents choose to continue wearing them postpandemic, even when it is no longer mandatory. This study aims to explore adolescents' experiences of wearing face masks during the COVID‐19 pandemic when their use was no longer mandatory.

**Method:**

In this exploratory qualitative study, data were collected from 16 adolescents through face‐to‐face semistructured interviews. The participants were asked about the reasons and feelings associated with continuing to wear masks, as well as the contexts in which they felt safe without a mask. The collected data were analysed using Braun and Clarke's thematic analysis.

**Results:**

Three main themes were identified: (1) Navigating complex decision‐making: balancing safety and self‐image, (2) peer influence dynamics and (3) managing the future: weather dynamics and pandemic evolution.

**Discussion:**

The potential implications of withdrawing COVID‐19 preventive measures, such as mask‐wearing, beyond the pandemic remain understudied. It is crucial to further investigate the perceptions related to wearing masks and its cessation, especially amongst vulnerable individuals.

**Patient or Public Contribution:**

Due to methodological constraints associated with participants' age, they were not engaged in the design, data analysis, data interpretation or manuscript preparation phases of the study.

## 1 INTRODUCTION

In response to the COVID‐19 pandemic, various measures were implemented to control the spread of the virus, including social distancing and mask‐wearing.^1^ Social distancing, a health practice that was frequently scrutinised,^2^ and mask‐wearing, which was deemed an essential preventive measure, were both widely enforced in many countries.^3^ During the postpandemic period of COVID‐19, the practice of wearing masks was maintained.^4^ Wearing a mask can help prevent contagion by reducing the spread of virus‐loaded droplets, particularly in situations where social distancing is not possible.^5^ Epidemiologic studies have shown that mandatory use of masks is associated with a reduction in COVID‐19 cases at the community level.^6^

As part of the action plan for the 2020–2021 school year in Catalonia, Spain, all educational stages began with 100% in‐person attendance. Full attendance was only partially reduced in the postmandatory stage between November 2020 and April 2021. Regarding personal prevention measures in educational centres, the main measures were physical distancing and the use of masks. The use of masks in educational centres was mandatory in the 2021–2022 course to prevent the spread of COVID‐19.^7^ However, on 19 April 2022, the requirement for masks in all internal spaces was eliminated.^8^

According to Goldstein and Flicker,^9^ research indicates the significance of examining the impact of COVID‐19 restrictions on adolescents, including the effects of deteriorated relationships on their mental and emotional health. Several studies have estimated the frequency of face mask use amongst young people, and approximately half of the participants always wore a face mask or covering in public.^10^, ^11^ Most studies examining the impact of the pandemic or mask‐wearing have focused on the period when mask‐wearing was mandatory or on the reasons for and facilitators of adhering to the regulations.^12^ Research into face mask adherence in the pandemic COVID era reveals intriguing gender‐based disparities and in most cases is inconclusive. Studies by Galasso et al.^13^ highlight differing compliance rates between genders, indicating that females tend to exhibit higher adherence to mask‐wearing protocols compared to males. Additionally, research by Howard et al.^12^ highlights the influence of gender‐specific attitudes and perceptions towards mask usage, attributing these differences to varying social norms and perceived susceptibility to viral transmission. However, there is limited knowledge regarding adolescents' experiences with COVID‐19 or their use of face masks in the postpandemic period. No study has yet described the factors that influence adolescents to use face masks, even when mask‐wearing is no longer mandatory in many countries. This study aims to explore Catalan adolescents' experiences of continuing to wear face masks despite the absence of a mandate.

## 2 METHOD

This study is based on the COVID‐19 Sentinel Schools Network of Catalonia.^14^ Its main objective was to monitor and evaluate the epidemiological situation of COVID‐19 and its determinants in the educational setting. The aim was to gather evidence for health policies aimed at preventing and controlling severe acute respiratory syndrome coronavirus 2 infection in schools. The study utilised an exploratory qualitative design, which enables a thorough comprehension of a particular phenomenon within a specific context and is suitable for investigating less‐known phenomena.^15^ The Ethical Committee of the Foundation University Institute for Research in Primary Health Care Jordi Gol i Gurina approved it on 17 December 2020 (code 20/192‐PCV).

Study participants were students aged 12–17 years, selected from two high schools in Catalonia. The only inclusion criterion was the wearing of a mask during the removal of restrictions, and they were recruited by the research team in coordination with their educational institution. Participants who were 16 years or older received written and oral information about the study and provided signed informed consent before the interviews. For participants under 16 years old, their parents provided signed informed consent after they were informed about the study. The data were collected by means of face‐to‐face semistructured interviews, which allowed for the collection of personal information in a guided and trustworthy manner.^15^ Sixteen individuals aged 12–17 from two different sociocultural contexts (eight girls and eight boys) were interviewed in May 2022. This sample size was considered sufficient to achieve information saturation.

The interview script was developed based on a prior study^16^ and the research interests. It addressed the following topics: reasons for continuing to use face masks, emotions associated with their use and situations in which individuals felt safe without wearing a mask. All interviews were conducted by a female researcher with a PhD and extensive experience in a private and secure location within an educational centre. All participant identities were kept anonymous, and a pseudonym was used throughout the interviews and transcriptions. The transcripts were securely stored on the university campus and protected with a security key, with access limited to the research team.

The collected data were analysed using Braun and Clarke's thematic analysis,^17^ which consists of six steps: (1) becoming familiar with the data, (2) coding the data, (3) categorising the data, (4) reviewing the categories, (5) defining and naming the categories and (6) writing the report. To increase the credibility and consistency of the results, two researchers (M. A.‐G. and J. M. L.‐M.) performed this procedure in parallel. Additionally, Atlas.ti® software was utilised to manage and analyse the textual data, providing increased rigour to the procedure.

Recommendations for Establishing Validity in Qualitative Inquiry^18^ were applied throughout the process. Consensus meetings were held with two expert researchers, and regular discussions and debriefing sessions were conducted concurrently. Reflective and critical thinking guided the entire process, providing robustness to the findings. Each data analyst reviewed the codes, themes and interview quotations separately, and all disagreements were resolved through discussion. Finally, the findings were discussed in three sessions with five experts, including two epidemiologists and three nurses, all with previous experience in qualitative methods. No changes were required after the discussions.

## 3 RESULTS

The thematic analysis identified three main themes: (1) Navigating complex decision‐making: balancing safety and self‐image, (2) peer influence dynamics and (3) managing the future: weather dynamics and pandemic evolution.

### 3.1 Navigating complex decision‐making: Balancing safety and self‐image

The discourse with these adolescents uncovered layers of uncertainty intertwined with their decision‐making process. The prevalent indecision amongst the participants underscored a profound hesitancy, indicative of a complex psychological landscape not solely governed by the information disseminated by educational entities. This prolonged uncertainty suggested deeper psychological considerations beyond the explicit instructions received, suggesting a nuanced journey towards adaptation.

It's a tough call, you know? Like, I'm totally over these face masks, but then again, I really don't wanna deal with COVID. Honestly, I'm just not feeling ready. I need more deets, you know? Gotta be sure about what I'm getting into before I make a move. (Male, 15 years old)

The qualitative exploration delved into the subtle reasons driving the sustained usage of face masks, even after the official revocation of mandatory measures. Safety concerns were prominently interwoven with the fabric of these adolescents' decisions, indicating a deep‐rooted apprehension about potential risks. Additionally, the preservation of self‐image emerged as an intricate factor, illuminating the role of masks in fortifying their self‐perception and identity amidst evolving societal norms. Thematic analysis unveiled a multifaceted narrative, emphasising the intricate interplay between psychological readiness, institutional communication and individual motivations that shape the adolescents' ongoing engagement with face masks beyond the cessation of compulsory mandates.

#### 3.1.1 Feeling safe

Amidst the fabric of continued mask usage, a profound narrative emerged centring on the bedrock of security. Participants consistently conveyed that the essence of security woven into mask‐wearing transcended mere protection against infection. These masks, a ubiquitous accessory, became emblematic of a shield, a safeguard against the invisible threats lurking in daily interactions. They were not merely perceived as a preventive tool against infection or reinfection; they encapsulated a broader sense of fortification, a tangible manifestation of reassurance in navigating the uncertainties of everyday life.

It's easy, you know? I just feel way safer this way. I don't wanna get sick or make my folks sick. My mask is like my shield, keeps me all protected and stuff. (Male, 13 years old)

The significance of masks extended beyond personal safety; it radiated into familial orbits. Within this tapestry of security, participants elucidated how these masks took on a role far beyond their physical presence. They emerged as symbols of care and responsibility, serving as a barrier, a guardian protecting beloved parents, revered grandparents and vulnerable loved ones from potential contagion. This layer of security woven into the fabric of mask‐wearing underscored a deeply ingrained ethos of safeguarding the cherished, a sentiment intricately interwoven into the motives for its continued use.

Got two big reasons I stick to masks. One, the virus ain't gone, just not as gnarly. And two, I hang with a bunch of older folks. Don't wanna risk getting them sick. Masks give me peace and let me kick it with them without stress. (Female, 14 years old)

Embedded within the adolescents' mask‐wearing decisions lay a complex tapestry of influences and personal convictions. Their choices were intricately shaped by the voices of authority figures like parents and teachers, whose opinions held significant sway in their decision‐making. For these adolescents, wearing masks transcended a mere health measure; it was a multifaceted necessity intertwining their biological, emotional and social equilibrium, all rooted in their unique life circumstances. Despite governmental directives urging mask removal, these adolescents exhibited a resolute rejection, showcasing an adaptive stance towards their immediate surroundings. Their perseverance in mask usage was not solely a rebellion against directives; rather, it signified a fusion of personal comfort and environmental adjustment. The divergence in appearance or social conformity did not discourage them; instead, their comfort stemmed from the multifaceted benefits masks offered in safeguarding their wellbeing, nurturing a sense of ease rooted in their individual reasons.

I can't really put it into words. Wearing it just makes me feel better, you know? I get that I'll ditch it someday, but I gotta feel like I'm cool with it first. I don't wanna be all out there or rebellious; I just wanna do it when I'm good and ready. Why rush it, you know? (Female, 13 years old)

Central to their steadfastness was an unwavering sense of security that masked their decisions. This feeling of safety was not merely a notion but a validated validation—a stamp of approval reinforcing their choice to persist with masks beyond the mandate's end. This security served as a validating force, affirming their decision to adhere to a practice deemed essential for their holistic wellbeing.

At the moment, I'm vibing with this [wearing the face mask]. It's all about feeling chill and safe. I'll use it for a couple more weeks and then we'll see how things go. (Male 12 years old)

#### 3.1.2 Protecting my self‐image

Within the data emerged a nuanced portrayal of how face masks served a dual purpose: as a shield against infections and as a veiled shelter concealing more than just physical vulnerabilities. Beyond their protective function, masks became a canvas for masking not only infections but also concealing facial expressions and various skin conditions like eczema, acne and other lesions. Interestingly, this concealment emerged as a prominent benefit, notably amongst girls below the age of 14, accentuating its impact on their social dynamics and self‐image within their peer groups.

It's been like forever, you know? Not dolling up, not stressing about how I look. Now, I'm just not feeling it, not feeling quite ready. (Female, 15 years old)

For these young girls, the mask represented more than a barrier against contagion; it held an unspoken power to navigate the intricacies of social belonging. Wearing a face mask was more than a health measure; it became a tool for meeting the social demands and expectations within their circles. It enabled them to harmonise with the norms of their group, shielding not just their health but also their social relevance and integration within their adolescent spheres. This qualitative exploration sheds light on the multifaceted role masks played, beyond mere physical protection, unveiling their intricate intersection with social dynamics and self‐perception amongst young girls.

Wearing my mask adds a layer of security. It's like my shield, you know? It hides my expressions, like my smile or my nose. When I see peeps taking off their masks, they seem so different, and I'm not into that vibe. I'm not sure if I look weird without it, but I'd rather keep it on for that extra safety vibe. It bugs me how we've gotten used to seeing each other like this. (Female, 16 years old)

### 3.2 Peer influence dynamics

Within the context of shifting mandates surrounding face coverings and the broader societal adjustments, adolescents stand within a nexus of influences, where two dominant forces, namely parental guidance and peer pressures, exert profound impacts on their decisions pertaining to the removal of masks. This juxtaposition of influences engenders a dynamic tension within the adolescent psyche, juxtaposing the nurturing encouragement from parental figures, emphasising safety and prudence, against the subtle yet compelling influence exerted by peer groups advocating for the adoption of unmasked social norms.

#### 3.2.1 Parents support

The significance of family dynamics in the decision to wear masks was critical. Adolescents reported receiving positive reinforcement from their parents for their decision to continue wearing masks. According to the adolescents, parents consistently showed respect and empathy towards their children's commitment to wearing masks, creating a supportive environment for decision‐making within the household.

I always feel supported by my parents in all my decisions. They even support me in my decision of whether or not to wear a mask at home, although I think they know I'm tired of using it. (Female, 13 years old)

While adolescents reported that their parents were aware of their interactions in social settings such as schools and recreational environments, they also reported that their parents consistently empowered them to independently manage their mask usage, particularly within their own home. This approach encourages self‐determination regarding mask‐wearing, promoting an environment that fosters agency and personal responsibility. It aligns with adolescents' need for independence in their decision‐making process.

I remember my mother saying, ‘I would not use it anymore If I were you, but you are old enough to decide’. She respected me. (Male, 15 years old)

#### 3.2.2 Peer pressure

In the context of family support and individual decision‐making, certain adolescents found themselves compelled to relinquish their masks, not by personal decision but due to insistent pressures and commentaries from their peer circles or educators. This intrusion of external influence altered their trajectory, pushing them away from their individual decisions towards mask removal. Such moments, where adolescents felt coerced to shed their masks against their preferences, ushered in an intensified realm of social pressures, tangibly altering their social dynamics and personal comfort zones. This shift from autonomous decision‐making to yielding to external pressures emphasised a nuanced layer within their experiences, exposing the intricacies and vulnerabilities interwoven into their interaction with the external world.

Some folks said I could ditch the mask, but I felt it was better to stick with it for me and my buddies. There was this one classmate who kept wearing theirs even after they said we didn't have to, but then the teachers asked her to take it off, and she did. It was kinda weird when I took mine off, felt like stripping down. Hard to put into words, you know? (Male, 8 years old)

In the complex network of adolescent behaviours related to wearing masks, certain spaces emerged as havens where masks were willingly removed. Homes, school playgrounds and eateries provided a sense of comfort and refuge, creating an environment where people could let their guard down and remove their masks. The allure of these spaces lay not just in their physical attributes but in the intangible sense of safety they offered. Adolescents found solace in brief periods spent within these realms, attributing their comfort to factors beyond mere physical settings.

I ditch my mask when I grab breakfast at the playground break. It stays off till I hit the classroom. I'm more freaked about catching the virus inside than outside. (Male, 14 years old)

### 3.3 Managing the future: Weather dynamics and pandemic evolution

During the postpandemic period, adolescents faced a complex interplay of emotions and considerations when deciding whether to continue wearing face masks despite eased restrictions. Emerging themes shed light on their nuanced experiences, with prevailing feelings centring around a hopeful anticipation for better weather conditions as a potential catalyst for adjusting mask‐wearing behaviours. This expectation intertwines with a palpable distrust concerning the evolving trajectory of the pandemic, weaving a tapestry of cautious uncertainty. Adolescents displayed a delicate balance, fluctuating between a desire for brighter, outdoor‐oriented seasons and a lingering caution due to the unpredictable course of the pandemic. These themes emphasise the intricate interplay of weather‐related hopes and persistent scepticism as adolescents grapple with the decision to persist with face mask usage beyond regulatory relaxations.

#### 3.3.1 Waiting for the summer

In contemplating future scenarios, adolescents exhibited an acute awareness of the impending cessation of mask usage, foreseeing a potential shift during the summer months or holiday periods. Their projections centred around envisaging a mask‐free phase, particularly during seasonal activities like water sports, frolicking in the sand or mingling with new acquaintances. Despite their evident hesitance in parting with face masks, these adolescents held a clear understanding that this practice was transient. They viewed it as a temporary safeguard, acknowledging its eventual discontinuation, foreseeing a gradual enhancement in safety measures as time progressed.

I might just remove my mask indoors this summer. It's been seriously suffocating in the heat lately. I'll either go mask‐free but be careful or just keep my distance from peeps at higher risk. (Male, 17 years old)

#### 3.3.2 Distrust about the evolution of the pandemic

In delving into the adolescent experience, a profound tapestry of apprehension and uncertainty unfolded, woven intricately into their perceptions of the pandemic's course and potential impositions, notably the looming reintroduction of face mask mandates. Their expressions revealed a deeper layer of distrust and disillusionment shrouding their perceptions of the pandemic's management. What surfaced amidst these narratives was an unsettling unease regarding the potential resurgence of masked existences and social distancing measures, particularly within the crucial juncture of their formative years. The unease was not just about following health protocols; it was an emotive struggle, resonating with the weight of disrupted social ties and the hazy contours of an uncertain tomorrow. These uncertainties cast a deep shadow, not only permeating their present wellbeing but also affecting their aspirations for the future. It was within this intricate web of uncertainties that their trepidations regarding the reappearance of pandemic‐driven restrictions are ensnared, highlighting the intricacy and depth of their experiences within this complex milieu.

Stuff's pretty chill and quiet these days, but it feels like it might not be done yet. I'm stressing about maybe having to mask up again, especially when it's time to party during the holidays. That would be a total bummer. And being away from my crew? Nah, that's a no‐go for me. (Male, 15 years old)

## 4 DISCUSSION

The study provides insight into the use of face masks beyond health behaviours, as mask‐wearing has become optional in many countries. The study demonstrates that perceptions of personal and familial vulnerability are the primary determinants of mask‐wearing. This finding is consistent with Martinelli et al.'s^19^ research, which also highlights the societal and personal dimensions of wearing masks, such as the perception of infection risk and a sense of responsibility and solidarity. Hanna et al.^20^ reported that the adult population believes that wearing masks provides personal protection and reduces the transmission of the virus as its primary benefit. Additionally, Warnock‐Parkes et al.^21^ found that the strongest predictors of wearing masks are an individual's belief in their ability to protect others and having an underlying health condition. Therefore, individuals' decisions to wear masks or not were influenced by their social connections, personal judgement and perceived susceptibility.^22^ These findings can be explained by Fineman's theory of vulnerability, which states that people are constantly exposed to multiple biopsychosocial hazards, making them vulnerable to harm and injury due to the ever‐present possibility of harm and injury.^23^ ^,p.9^

The study by Chen and Lei^24^ employed the Protection Motivation Theory (PMT) to understand adolescents' decisions regarding mask adoption. According to PMT, individuals' protective behaviours are influenced by their perception of the severity and susceptibility of a threat, as well as their belief in the effectiveness of the response (response efficacy) and their confidence in executing the required behaviour (self‐efficacy). In this context, adolescents demonstrated adherence to these PMT constructs, as their decision to continue wearing masks was substantiated by various factors. First, their awareness of the improved epidemiological situation did not diminish their belief in susceptibility; they maintained a perception that they or their family members could still fall ill. Second, their conviction in the effectiveness of masks in halting disease transmission remained firm, indicating a belief in the response's efficacy. Lastly, adolescents displayed confidence in their ability to consistently use masks (self‐efficacy). This alignment between the PMT constructs and adolescents' persistent mask use illustrates the applicability of PMT in explaining and predicting individuals' decisions to engage in protective behaviours like continued mask‐wearing.

Building on Chen and Lei's^24^ research, our study aims to contribute to the understanding of psychosocial factors that influence mask‐wearing behaviour in adolescents. Although adolescents were aware of the improved epidemiological situation, they also had a nuanced understanding of their vulnerability to infection. This statement emphasises the dynamic interaction between external circumstances and individual behavioural choices, enriching the applicability of the variables within the PMT framework. Research suggests that attitudes and policies towards mask use play a critical role in mask adoption and lead to higher adoption rates. Additionally, a more stringent face mask policy is correlated with a higher probability of mask usage.^25^ The findings of the present study suggest that face masks are used for both personal health protection and the protection of those around the wearer. Additionally, they may provide a sense of security in terms of physical appearance. Previous research has investigated the social representations and significance of wearing face masks during the pandemic.^19^, ^22^ These studies highlighted the various roles that face masks play in people's lives, extending beyond their primary purpose of protection.

Amongst the participants in this study, female individuals under the age of 14 reported wearing face masks due to self‐image reasons. During this stage of adolescence and identity development, self‐concept and personal perception are not the only influencing factors; the perceptions of others, particularly their peers, are highly relevant. Adolescents form crucial relationships with their peers in their search for self‐identity.^26^ Therefore, it is possible that adolescents are more concerned with the image they convey than with their actual emotions.^27^ It is worth noting that many adolescents who reported struggling with self‐image began their journey of identity formation while wearing a face mask. This may explain why adolescents may be reluctant to stop wearing masks, despite expressing a desire to do so. Research has shown that gender does not influence the decision to wear a face mask. However, it may affect perceptions of face masks. For example, women may perceive face masks as uncomfortable, while men may view them as a threat to their independence.^28^

Another reason why some adolescents were hesitant to stop wearing face masks is because of concerns about their self‐image and social anxiety. Social anxiety is a health condition in which an individual experiences fear or apprehension in social interactions, particularly those where they may be evaluated by others.^29^ The present study's results could be explained by Saint and Moscovitch's^30^ findings, which suggest that mask‐wearing can provide a sense of security, particularly for individuals dealing with social anxiety. The use of masks can act as a protective shield, reducing discomfort associated with being exposed to others and the possibility of negative evaluations in social situations. This protective function may have motivated the adolescents in the present study to continue wearing masks beyond the mandatory period.

This study has some limitations that should be taken into account. First, the data were collected in two different sociocultural contexts, but they are limited to the realities, imaginaries, policies and norms present in Barcelona. Consequently, the results may only be transferable (not generalisable) to adolescents in large Western European cities. Second, it is important to note that the study only interviewed participants who volunteered, which may have limited the scope of the experiences considered. Additionally, conducting interviews within schools may have introduced a social desirability bias, despite the interviewees not being affiliated with the educational process. Finally, the interviews were conducted and analysed in Spanish or Catalan. To ensure the accuracy and naturalness of the findings, the translation process was overseen by two bilingual researchers and professional translators proficient in both languages.

## 5 CONCLUSION

This study found that adolescents, mostly girls, continued to use face masks voluntarily during COVID‐19, even though their use was no longer mandatory, for fear of infecting their vulnerable relatives and to protect their self‐image. Parents and other family members played a key role in helping them make this decision and reinforcing it. Adolescents reported feeling vulnerable and anxious when not wearing a face mask in places they considered risky.

As previously mentioned, there is limited research on the withdrawal of COVID‐19 preventive measures, including mask‐wearing, and the potential implications of transitioning from wearing masks to not wearing them beyond the pandemic. Therefore, it is crucial to further investigate the experiences and perceptions related to both mask usage and discontinuation, especially amongst individuals in critical and sensitive stages, such as childhood and adolescence. Furthermore, it is important to examine individuals' attitudes towards those who opt to continue wearing masks after the pandemic, taking into account the possible social implications.

The study results emphasise the significance of investigating the effects and outcomes of implementing strict public health measures, such as those enforced during the pandemic. The findings add to the expanding body of research on mask‐wearing behaviour and offer valuable insights into public health interventions during pandemics. These findings could help inform the development of future policies and guidelines that more accurately reflect individuals' experiences and perceptions of mask‐wearing, by incorporating psychosocial perspectives into public health directives and addressing fears related to vulnerability and personal image in educational centres. More inclusive and responsive postpandemic guidelines could be developed by creating supportive environments in educational settings, prioritising mental health aspects and including youth perspectives in policy formulation.

## AUTHOR CONTRIBUTIONS

**Mariela Aguayo‐González**: Investigation; writing—original draft; writing—review and editing; formal analysis; data curation; conceptualisation. **Juan M. Leyva‐Moral**: Conceptualisation; validation; formal analysis; supervision; writing—review and editing; writing—original draft; methodology. **David Giménez‐Diez**: Conceptualisation; investigation; writing—review and editing; writing—original draft. **Andreu Colom‐Cadena**: Conceptualisation; writing—review and editing. **Isabel Martínez**: Conceptualisation; writing—review and editing. **Carolina Watson**: conceptualisation; writing—review and editing. **Anna Bordas**: Conceptualisation; writing—review and editing. **Cinta Folch**: Conceptualisation; writing—review and editing. **Jordi Casabona**: Conceptualisation; writing—review and editing.

## CONFLICT OF INTEREST STATEMENT

The authors declare no conflict of interest.

## ETHICS STATEMENT

This study was approved on 17 December 2020 by the Ethical Committee from the Institute for Research in Primary Health Care Jordi Gol i Gurina (IDIAPJGol) (code 20/192‐PCV).

## Data Availability

The data that support the findings of this study are available from the corresponding author upon reasonable request.
